# The efficacy and safety of irreversible electroporation for the ablation of renal masses: a prospective, human, in-vivo study protocol

**DOI:** 10.1186/s12885-015-1189-x

**Published:** 2015-03-22

**Authors:** Peter GK Wagstaff, Daniel M de Bruin, Patricia J Zondervan, C Dilara Savci Heijink, Marc RW Engelbrecht, Otto M van Delden, Ton G van Leeuwen, Hessel Wijkstra, Jean JMCH de la Rosette, M Pilar Laguna Pes

**Affiliations:** 1 Department of Urology, Academic Medical Center, Meibergdreef 9, 1105AZ Amsterdam, Netherlands; 2 Department of Pathology, Academic Medical Center, Meibergdreef 9, 1105AZ Amsterdam, Netherlands; 3 Department of Radiology, Academic Medical Center, Meibergdreef 9, 1105AZ Amsterdam, Netherlands; 4 Department of Biomedical Engineering & Physics, Academic Medical Center, Meibergdreef 9, 1105AZ Amsterdam, Netherlands; 5 Department of Electrical Engineering, Eindhoven University of Technology, Den Dolech 2, 5612 AZ Eindhoven, Netherlands

**Keywords:** Irreversible electroporation, IRE, Ablation, Kidney, Renal mass, Cancer, Safety, Efficacy

## Abstract

**Background:**

Electroporation is a novel treatment technique utilizing electric pulses, traveling between two or more electrodes, to ablate targeted tissue. The first in human studies have proven the safety of IRE for the ablation of renal masses. However the efficacy of IRE through histopathological examination of an ablated renal tumour has not yet been studied. Before progressing to a long-term IRE follow-up study it is vital to have pathological confirmation of the efficacy of the technique. Furthermore, follow-up after IRE ablation requires a validated imaging modality. The primary objectives of this study are the safety and the efficacy of IRE ablation of renal masses. The secondary objectives are the efficacy of MRI and CEUS in the imaging of ablation result.

**Methods/Design:**

10 patients, age ≥ 18 years, presenting with a solid enhancing mass, who are candidates for radical nephrectomy will undergo IRE ablation 4 weeks prior to radical nephrectomy. MRI and CEUS imaging will be performed at baseline, one week and four weeks post IRE. After radical nephrectomy, pathological examination will be performed to evaluate IRE ablation success.

**Discussion:**

The only way to truly assess short-term (4 weeks) ablation success is by histopathology of a resection specimen. In our opinion this trial will provide essential knowledge on the safety and efficacy of IRE of renal masses, guiding future research of this promising ablative technique.

**Trial registration:**

Clinicaltrials.gov registration number NCT02298608.

Dutch Central Committee on Research Involving Human Subjects registration number NL44785.018.13

## Background

The past two decades have shown a steady increase in the incidence of small renal masses (SRM) up to 4 cm [1,2]. Nephron sparing surgery, in the form of partial nephrectomy, is considered to be the gold standard for treatment of SRMs [3]. Currently thermal focal therapies such as cryoablation and radiofrequency ablation (RFA) are primarily recommended in patients who are poor surgical candidates or have a genetic predisposition for developing multiple tumours [4-6]. However, promising long-term results combined with little or no loss in renal function have created interest in thermal focal therapies as a future treatment option for a broader range of patients [7-10].

Focal treatment of kidney tumour requires precisely dosed and accurate targeting of the tissue to be ablated while preserving surrounding healthy tissue and vital structures such as blood vessels, nerves, the renal collecting system and neighbouring organs [11]. The unselective destruction of currently practiced thermal ablation techniques can result in damage to vital structures in the vicinity of the tumour and undesired excessive ablation of normal parenchyma [12]. Thermal ablation intensity can be impaired due to ‘heat sink’ in the vicinity of large vessels and the renal collecting system [4].

Electroporation or electropermeabilisation is a technique in which electric pulses, traveling between two or more electrodes, are used to create ‘nanopores’ in the cell membrane. These pores allow for molecules to pass into the cell. The process can be temporary (reversible electroporation, RE), however above a certain threshold the ‘nanopores’ become permanent causing cell death due to the inability to maintain homeostasis (irreversible electroporation, IRE) [13-15]. It has been hypothesized that IRE is not dependent on temperature and is therefore not influenced by ‘heat sink’ promising consistent ablation results [11]. In theory IRE is defined to damage of the cell membrane, sparing tissue architecture and minimizing damage to blood vessels, nerves and the renal collecting system [16]. Recent literature however, has predicted [17] and measured [18,19] a large increase of temperature in healthy porcine kidney using currently practiced equipment and settings. As a result, it remains unclear to which extent the thermal effect or the electroporation contribute to the IRE ablation effect. Histopathology using viability staining of renal IRE lesions shows a sharp demarcation between ablated and non-ablated tissue allowing for precise targeting while sparing the surrounding healthy tissue [20,21].

Animal trials have assessed the use of MRI and CT imaging for the intermediate follow-up of IRE lesions. Contrast enhanced CT imaging directly after IRE ablation of porcine kidney showed a hypodense non-enhancing lesion, persisting at 1 week post IRE. At 3 weeks, 4 out of 6 IRE lesions had disappeared completely [22]. Thomson et al. performed in human IRE in 10 renal tumours with subsequent follow-up by CT imaging. At 3 months post IRE incomplete ablation was diagnosed in 2 patients on the basis of CT-imaging. However, the authors provide little information on the imaging characteristics of the residual lesion. MRI directly after IRE of porcine kidney showed a localized oedema at the region of IRE ablation. At 7 days after IRE a hypo-intense necrosis-like lesion in the renal parenchyma at the region of IRE was visualised. Finally, at 28 days a sharply delineated, non-intense, scar-like lesion with cortical shrinkage and without contrast enhancement was visualised [20]. These results provide an insight in the use of imaging for the follow-up of renal IRE. However, a study where follow-up imaging, specifically assessment of ablation volume and residual enhancing tumour, is correlated to histopathology of the resected specimen has not yet been performed.

Procedural safety of renal IRE in humans has been tested and confirmed [15]. The electric pulses administered during IRE have the potential of causing cardiac arrhythmias; by synchronising the IRE pulses with the ECG this complication can be avoided [23]. In a study by Pech et al., ablated tumours were resected directly after IRE and they observed swelling of cells but no actual cell death. However, histological staining to assess cell viability was not performed [15].

Before progressing to a long-term IRE follow-up study it is vital to have pathological confirmation of the efficacy of the technique. Furthermore, follow-up of IRE ablation requires an accurate imaging modality. This trial will investigate IRE ablation efficacy by correlating 3D histopathology of a resected IRE lesion with: 1) 3D reconstructed imaging using MRI and contrast enhance ultrasound (CEUS), and 2) the 3D predicted ablation volume as provided by the manufacturer. The objectives of the study are assessing the safety and efficacy of IRE of renal masses (primary objectives), and assessing the efficacy of MRI and CEUS for the initial evaluation and short-term (4 weeks) follow-up of IRE lesions (secondary objectives). This study conforms to the recommendations of the IDEAL Collaboration and can be categorised as a phase 2A development trial [24].

## Methods/Design

### Ethical consideration

The Institutional Review Board (IRB) of the Academic Medical Center, Amsterdam, approved this study protocol (2013_219). The protocol has been registered with The Dutch Central Committee on Research Involving Human Subjects (NL44785.018.13) and is entered in the clinicaltrials.gov database (NCT02298608). Potential candidates will receive the study information both verbally and in writing. They will be granted at least one week to decide on participation. Written informed consent is acquired from all participants.

### Study design

**Figure 1:**
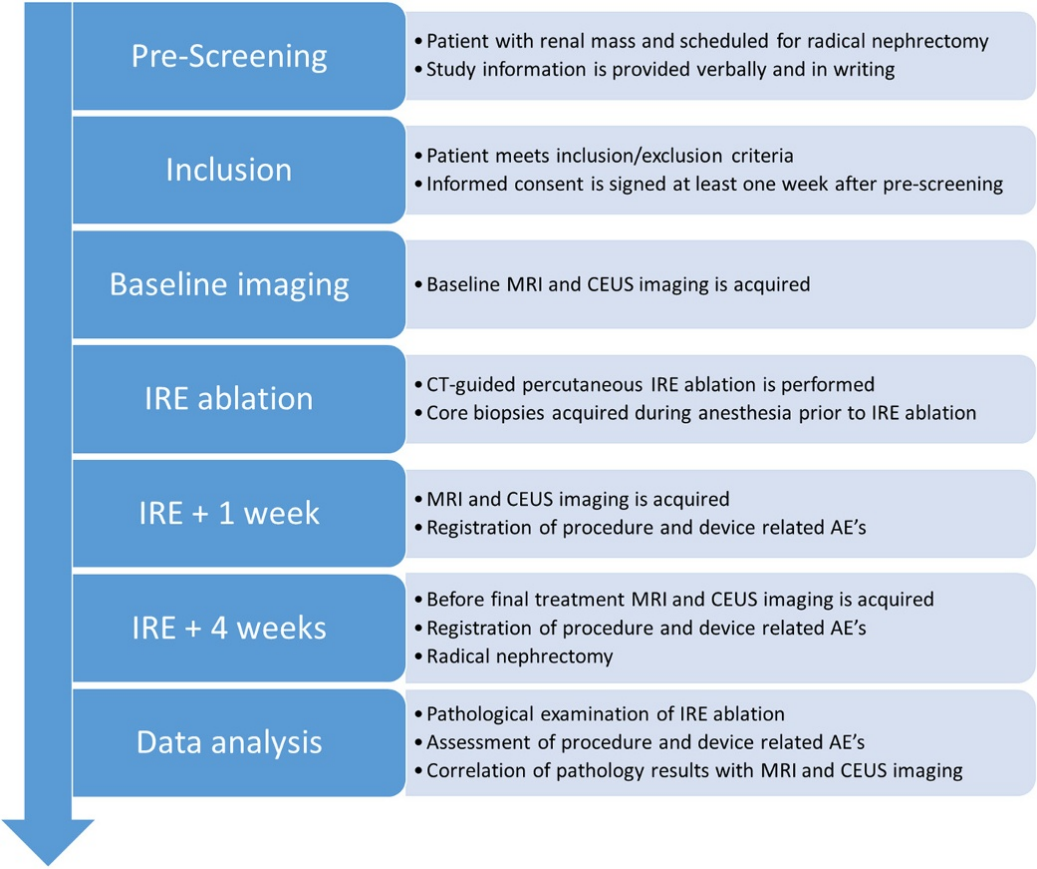
**Study design flowchart.**

This is a prospective, human, in-vivo study among 10 patients presenting with a solid renal mass, and candidates for radical nephrectomy (RN). A study flowchart is provided in Figure 1. Prior to the IRE procedure baseline MRI and CEUS imaging will be acquired. Subsequently the patients will undergo IRE ablation of their renal mass. Follow-up at one and four weeks post IRE will be performed using MRI and CEUS imaging. At these time points procedure and device related adverse events (AE) will be registered using the Common Terminology Criteria for Adverse Events (CTCAE) version 4.0 guideline. Four weeks after IRE the patients will undergo radical nephrectomy after which pathological examination will be performed to evaluate IRE ablation success. Core biopsies are harvested before IRE ablation in order to assure tumour differentiation in case of complete ablation. Correlation between pathology and imaging will reveal the efficacy of MRI and CEUS for the assessment of IRE lesions.

### Study objectives

Primary objectives:

- To determine the efficacy of IRE ablation of renal masses, measured by pathological examination of the targeted tumour.
- To determine the safety of IRE ablation of renal masses, by evaluating device and procedural adverse events using CTCAE v4.0.

Secondary objectives:

- To evaluate the efficacy of MRI in the imaging of ablation success, the extent of the ablation zone, one and four weeks post IRE ablation.
- To evaluate the efficacy of CEUS in the imaging of ablation success, the extent of the ablation zone, one and four weeks post IRE ablation.

#### Population

Ten patients with a solid enhancing renal mass and scheduled for a radical nephrectomy will be enrolled in this study. Eligible patients are over 18 years of age, and candidate for radical nephrectomy due to tumour size/stage, or ESRD (stage 4 or 5), or the need for a pre-emptive transplant kidney. The development of IRE is aimed at the ablation of small renal masses (SRM). According to EAU and Dutch Association of Urology (NVU) protocol the preferred treatment of a SRM in an otherwise functioning kidney is partial nephrectomy (PN). Performing IRE ablation in these cases might, however complicate a subsequent partial nephrectomy leading to impaired surgical outcomes. Therefore we will strictly include patients with a renal mass who are planned for radical nephrectomy. All inclusions will be reviewed for safety and eligibility by a nephrologist participating in the research project. The inclusion and exclusion criteria for this study are listed in Table 1.

**Table 1:** **Inclusion and exclusion criteria**

Inclusion criteria	Exclusion criteria
○ Age ≥ 18 years	○ Irreversible bleeding disorders
○ Solid enhancing mass on cross sectional imaging	○ Inability to stop anticoagulation therapy
○ Scheduled for open or laparoscopic RN	○ Prev. cryoablation, RFA or PN affected kidney
○ Signed informed consent	○ Anaesthesia Surgical Assignment (ASA) cat. ≤ IV
	○ ICD or pacemaker
	○ Severe cardiovascular disease

Severe cardiovascular disease is defined as the diagnosis of myocardial infarction, uncontrolled angina, significant ventricular arrhythmias, stroke or severe cardiac failure (NYHA class ≥ III) within 6 months prior to inclusion.

#### Study procedures

##### Core biopsy (standard treatment)

Percutaneous renal core biopsy will be performed directly before the IRE procedure, utilizing the procedural anaesthesia. A minimum of two percutaneous core biopsies will be harvested for pathological examination. In the Academic Medical Center Amsterdam all patients presented with a renal mass on cross-sectional imaging, suspicious for malignancy, are advised to undergo renal core biopsies.

##### IRE ablation (study intervention)

**Figure 2:**
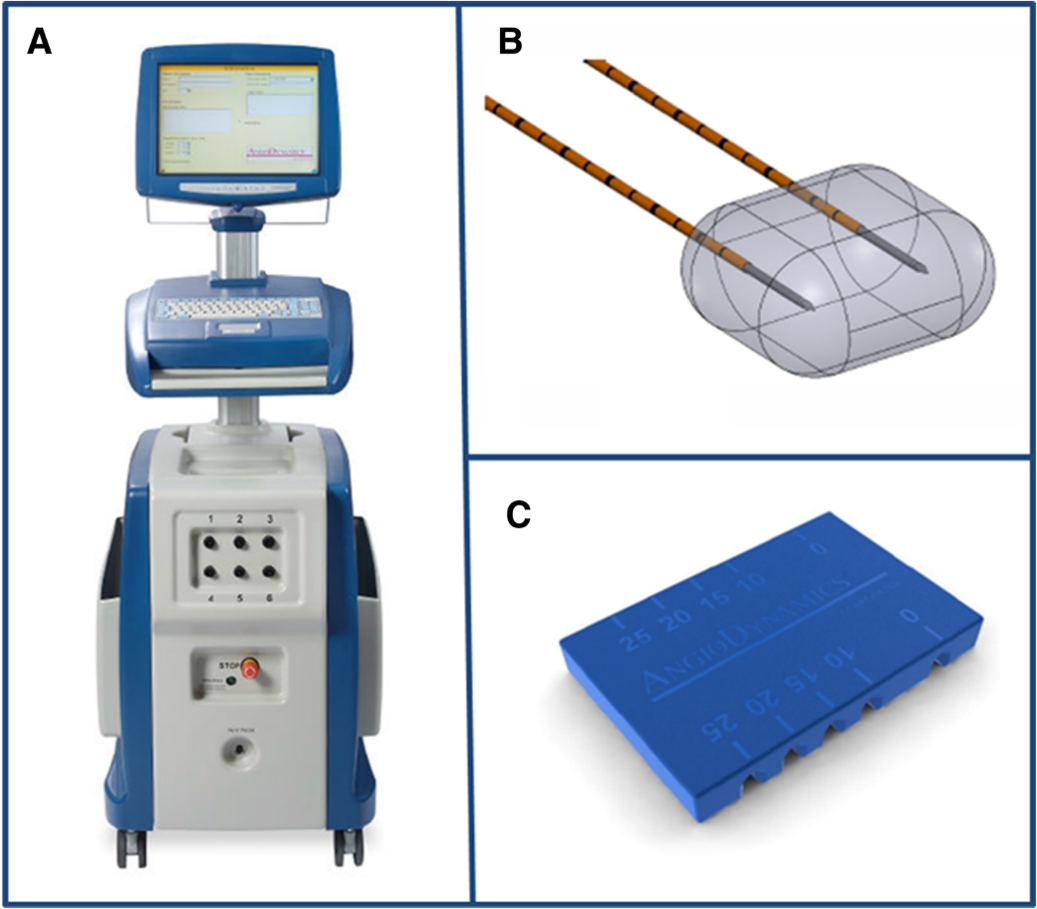
**IRE equipment.** The NanoKnife IRE console **(A)** utilizes 19G monopolar needle electrodes **(B)** which can be locked together using external spacers **(C)**.

This study utilizes the Angiodynamics (Queensbury, New York) NanoKnife™ IRE device (Figure 2A), also registered as the HVP-01 Electroporation System. This IRE system consists of a Low Energy Direct Current (LEDC) generator, footswitch and 19G monopolar needle electrodes (15 or 25 cm length). The device and electrodes have been developed for soft tissue ablation. Both the device and the electrodes carry a CE certificate for cell membrane electroporation. The system has been approved by the FDA via 510(k) Premarket Notifications (K060054, K080202, K080376, K080287). All 510(k) cleared components are indicated for surgical ablation of soft tissue.

The IRE procedure will take place at the Radiology department CT-room under general anaesthesia with muscle relaxation, as described by Nielsen et al. [25]. An interventional radiologist accompanied by a urological surgeon will perform the procedure. ECG monitoring and synchronization of IRE pulses will be performed under anaesthetic supervision. Needle electrodes (Figure 2B) will be placed under ultrasound and CT guidance using external spacers (Figure 2C) for fixation during pulse administration. Probe number and placement will be adjusted for specific tumour size or targeted tissue ablation in case of clinical tumor stage ≥ cT1b. Currently practiced IRE settings for tumour ablation are electrode spacing of 15 mm, electrode tip exposure of 15 mm, 90 pulses of 90 μs (synchronised with ECG) and a pulse intensity of 1500 V/cm. The IRE treatment cycle will take 5–10 minutes; total operating time is estimated at 90 minutes. If deemed clinically fit, patients will be discharged 24 hours after the IRE procedure. Post procedural pain will be quantified at 4 hours, 24 hours and 1 week by means of VAS score, cumulative opiate use and patient satisfaction.

##### CEUS and MRI imaging (study intervention)

CEUS and MRI imaging will take place at baseline, one week and four weeks after IRE ablation in order to assess lesion size and enhancement. Furthermore, this will reveal possible complications and any unexpected abnormalities as a result of the IRE procedure that may affect the final surgery (radical nephrectomy).

Contrast enhanced ultrasound (CEUS) utilizes a contrast agent to increase echogenicity of blood for better visualisation tissue vascularisation. Ultrasound contrast contains 3–5 μm microbubbles surrounded by a phospholipid shell. Early studies have shown promising results for the use of CEUS in the follow-up after cryoablation [26]. This study uses a Philips iU22 (Philips Healthcare, Bothell, USA) ultrasound device which is optimised for contrast studies, in combination with SonoVue (Bracco, Milan, Italy) a third generation ultrasound contrast agent with a elimination half-life of 6 min [27].

MRI will be performed using a Siemens Avanto 1.5 Tesla MRI scanner (Siemens Healthcare, Erlangen, Germany) with a 16-channel body array coil. The MRI protocol will include at least the following sequences: T2-trufi with fat suppression, T1-fl2d contrast enhanced in and out of phase, T2-haste, T1 vibe unenhanced and dynamic series at 30 seconds, 60 seconds, and 15 minutes. As MRI contrast agent Gadovist 1.0 (Bayer Pharma, Leverkusen, Germany) will be used.

##### Radical nephrectomy (standard treatment)

Radical nephrectomy will be performed four weeks after renal IRE ablation, either open or laparoscopic depending on patient specific factors such as co-morbidities and tumour characteristics. It will be performed according to department protocol by two experienced urologic surgeons.

#### Sample size

This is a phase 2A (IDEAL), pilot study. In ablation of renal masses, location and size of the renal mass influence treatment variables: number of probes and device settings. The sample size of 10 patients was chosen in order to explore 2–3 probe configurations. In this phase of research, this requires at least 3 repetitions of a specific probe configuration in order to assess consistency and the potential influence of other factors (tumor location, tissue composition) on ablation volume. Furthermore, a recently published animal study by Sommer et al. demonstrated a successful evaluation of CT imaging versus histological analysis with 3 probe configurations using a sample size of 10 cases [28]. A sample size of 10 patients, testing 2–3 IRE probe configurations, does not allow for reliable statistical analysis. We will therefore confine our results to averages and standard deviations of the assessed volumetric data.

#### Potential benefits and risks

There are no benefits for patients that participate in this study. Study participants will be exposed to additional risk when compared to standard treatment. They will have to undergo an additional procedure under general anaesthesia with muscle relaxation. An independent expert, assigned by the IRB, has estimated the exposure to ionizing radiation during the IRE procedure at 32 mSv.

IRE is a new tissue ablation technology and IRE of renal tumours has only been tested in a limited number of patients. It might be that certain risks and side effects are unforeseen at this point in time. Potential risks associated with IRE for renal tumours, using the NanoKnife™ system, are listed in Table 2. In addition, it is not expected that renal IRE in patients with ERDS will result in an acute dependence on dialysis. Both animal and human studies did not show a substantial decrease in GFR following renal IRE ablation. Research among patients suffering from renal insufficiency has however not yet been conducted. Therefore the possibility of a decrease in renal function leading to the need for dialysis cannot be completely excluded.

**Table 2:** **Potential risks associated with IRE of renal tumours**

Potential hazards of renal IRE ablation	Potential effects
Excessive energy delivery	Muscle contraction, burn, damage to critical anatomical structure, unintended tissue ablated, bradycardia/hypotension, vagal stimulation/asystole, electrical shock, myocardial infarction, stroke, death
Insufficient/no energy delivery	Ineffective ablation, no ablation
Unintended mains or patient circuit voltage exposure to patient or user	Electrical shock
Incorrect timing of pulse delivery	Transient arrhythmia, prolonged arrhythmia, stroke, death
Unintended interference with implanted devices containing electronics or metal parts	Myocardial infarction, stroke, death
Unexpected movement of the device and displacement of the electrodes	Hypotension, damage to critical anatomical structure, pneumothorax, mechanical perforation, haemorrhage, unintended tissue ablated, electrical shock, death
Sterile barrier breach	Infection, sepsis

#### Data safety monitoring board

The study will be monitored by a data safety monitoring board (DSMB) consisting of an independent urologist and a statistician. This team will monitor patient safety and treatment efficacy data during the study. Monitoring procedures are predetermined and described in the DSMB charter, approved by the IRB of the Academic Medical Center, Amsterdam. Additional DSMB meetings can be called at any time if deemed necessary by the DSMB or the Principal Investigator.

#### Analysis

The NanoKnife console provides 2D images displaying a cross section of the predicted ablation zone perpendicular to the needle tract. Using the AMIRA software package (FEI Visualisation Sciences Group, Burlington, USA) the 2D ablation zone cross sections will be stacked along the length of the exposed electrode tip providing predicted:

- 3D reconstruction
- ablation zone shape/symmetry
- ablation zone volume (cm^3^)

Pathological examination of the resected specimen will be performed by an experienced genitourinary pathologist. Prior to the study specific pathology protocol, sufficient material is acquired for routine renal tumour examination. The kidneys will be cut in a coronal plane creating 3–4 mm slices. After macroscopic inspection, the whole IRE lesion will be excised and embedded for sectioning and staining. Stains to be used will include hematoxylin and eosin (H&E), nicotinamide adenine dinucleotide (NADH) diaphorase and terminal deoxynucleotidyl transferase dUTP nick end labelling (TUNEL). NADH staining confirms cell viability and TUNEL staining conversely indicates cell non-viability. Combining the results of these stains will provide a detailed analysis of cell viability within the IRE ablation zone.

Microscopic examination will assess:

- IRE ablation volume (cm^3^)
- ablation zone shape/symmetry
- transition zone
- viable cells within ablation zone
- skip lesions
- damage to blood vessels
- damage to the collecting system
- damage to the renal pelvis

The pathology slides will be digitized using a Ventana iScan HT pathology slide scanner (Roche, Tuscon, USA). A 2D reconstruction of the segmented tumour will be constructed using Fiji (ImageJ). Within the 2D reconstruction the ablation zone will be outlined. Using the AMIRA software package the 2D tumour sections will be stacked to render a 3D reconstruction of the histopathology. This reconstruction is used to assess the exact lesion volume and shape.

MRI and CEUS imaging will be analysed by a specialised urologic radiologist focussing on:

- ablation volume (cm^3^)
- ablation zone shape/symmetry
- residual tumour on ablation zone border
- skip lesions within ablation zone
- transition zone between ablated and normal renal tissue
- damage to vital structures

Within the CEUS images basic measurements will be performed. Within in the MRI images the ablation zone and the kidney as a whole will be outlined. Using the AMIRA software package the outlined MRI images will be stacked to render a 3D reconstruction of the kidney and the IRE ablation zone within.

## Discussion

Before progressing to follow-up studies of IRE in renal masses it is vital to perform tissue specific testing of IRE ablation efficacy and safety. This trial will investigate IRE ablation efficacy by comparing 3D histopathological examination of a (partially) resolved IRE lesion, through radical nephrectomy with 1) examination of 3D imaging using MRI and CEUS and 2) 3D prediction of ablation volume as given by the manufacturer. IRE ablation volume and shape is influenced by many variables such as needle number, needle configuration, and device/pulse settings. With only 10 IRE ablations it is not within in the scope of this study to experiment with a wide variety of IRE settings. We aim to test 2 needle electrode configurations, while keeping the device settings constant. In clinical practice contrast enhanced CT scanning is most widely used modality for follow-up after renal mass treatment. In this study however it was decided not to investigate CT imaging in order to limit the cumulative radiation exposure. Study participants are already receiving an estimated 32 mSv of ionizing radiation due to the CT guided IRE procedure. Adding CT follow-up to the research protocol would result in 2–3 additional 4 phase CT scans, besides any CT scans that are necessary after the final treatment. Another limitation of this study is the follow-up period, which is limited at 4 weeks. Animal trials have shown renal IRE lesions to be partially resolved at 3–4 weeks [20-22]. Preferably radical nephrectomy would be postponed longer than 4 weeks, giving the IRE lesion more time to mature, allowing for better analysis of intermediate ablation results. However, further prolonging the final treatment is unethical at this early phase of the research. A final limitation is the tumour size. Patients who are candidate for radical nephrectomy, except for patients with ERDS, will have tumours larger than 4 cm. Ablative therapies are indicated for tumours ≤ 4 cm, which means that we not testing renal IRE in the intended population. The choice for radical nephrectomy was made out of the concern that IRE ablation might complicate a subsequent partial nephrectomy leading to impaired surgical outcome. In our opinion this trial will provide essential knowledge on IRE of renal masses, guiding future research of this promising ablative technique.

## Abbreviations

AE: Adverse event
CCMO: Central Committee on Research Involving Human Subjects
CEUS: Contrast enhanced ultrasound
CTCAE: Common terminology criteria for adverse events
DSMB: Data safety monitoring board
EAU: European association of urology
ESRD: End stage renal disease
GFR: Glomerular filtration rate
ICD: Implantable cardioverter-defibrilator
IRE: Irreversible electroporation
IRB: Institutional Review Board
PN: Partial nephrectomy
RFA: Radio frequency ablation
RE: Reversible electroporation
RN: Radical nephrectomy
SRM: Small renal mass
NVU: Dutch Association of Urologists
VAS: Visual analogue scale
